# Hierarchical genetic structure and implications for conservation of the world’s largest salmonid, *Hucho taimen*

**DOI:** 10.1038/s41598-021-99530-3

**Published:** 2021-10-15

**Authors:** Lanie M. Galland, James B. Simmons, Joshua P. Jahner, Agusto R. Luzuriaga-Neira, Matthew R. Sloat, Sudeep Chandra, Zeb Hogan, Olaf P. Jensen, Thomas L. Parchman

**Affiliations:** 1 Graduate Program in Ecology, Evolution, and Conservation Biology, University of Nevada, Reno, Reno, NV USA; 2 Department of Biology, University of Nevada, Reno, Reno, NV USA; 3 Wild Salmon Center, Portland, OR USA; 4 Global Water Center, University of Nevada, Reno, Reno, NV USA; 5 Center for Limnology, University of Wisconsin - Madison, Madison, WI USA

**Keywords:** Population genetics, Genetic variation, Conservation genomics

## Abstract

Population genetic analyses can evaluate how evolutionary processes shape diversity and inform conservation and management of imperiled species. Taimen (*Hucho taimen*), the world’s largest freshwater salmonid, is threatened, endangered, or extirpated across much of its range due to anthropogenic activity including overfishing and habitat degradation. We generated genetic data using high throughput sequencing of reduced representation libraries for taimen from multiple drainages in Mongolia and Russia. Nucleotide diversity estimates were within the range documented in other salmonids, suggesting moderate diversity despite widespread population declines. Similar to other recent studies, our analyses revealed pronounced differentiation among the Arctic (Selenge) and Pacific (Amur and Tugur) drainages, suggesting historical isolation among these systems. However, we found evidence for finer-scale structure within the Pacific drainages, including unexpected differentiation between tributaries and the mainstem of the Tugur River. Differentiation across the Amur and Tugur basins together with coalescent-based demographic modeling suggests the ancestors of Tugur tributary taimen likely diverged in the eastern Amur basin, prior to eventual colonization of the Tugur basin. Our results suggest the potential for differentiation of taimen at different geographic scales, and suggest more thorough geographic and genomic sampling may be needed to inform conservation and management of this iconic salmonid.

## Introduction

Population genetic data is relevant for shaping conservation, restoration, and management activities, and for understanding the response of populations to environmental change. Modern high throughput sequencing technologies have enabled genome-wide perspectives and improved our ability to quantify genetic variation across populations, including those of conservation concern^1,2^. Reduced representation approaches such as restriction site-associated DNA sequencing (RADseq) and genotyping-by-sequencing (GBS)^3^ have facilitated genome-wide population genetic analyses in organisms without genomic resources, and have often recovered patterns of fine-scale genetic structure and resolved patterns of recent diversification that were not evident with traditional molecular marker systems^4,5^. Such approaches have improved our understanding of patterns of population structure and connectivity^6,7^, the frequency and dynamics of hybridization^8,9^, and the potential for species response to environmental change^10,11^, and have guided the identification of conservation or management units^12,13^.

Globally, many salmonid fishes have experienced sharp declines, especially in recent decades^14^, due to anthropogenic factors including aquaculture^15^, introduced species, habitat degradation, overfishing, and climate change^16–18^. Population genetic data have been central to understanding evolutionary history of sensitive salmonid populations^19,20^ and for guiding their conservation and management^19,21^. High throughput sequencing in salmonids has improved the delineation of evolutionarily significant units^12^ and the detection of introgression between introduced and native populations^22,23^, as well as identifying the genetic basis of adaptive phenotypes^24–26^. These perspectives, however, have mostly been limited to certain regions where substantial funding has been allocated to bolster fisheries conservation and management, such as the North American Pacific Northwest (e.g., see Ref.^27^).

In contrast, salmonids occurring in sparsely populated regions of northern Asia have received comparatively little research attention. Despite hosting some of the world’s most isolated aquatic ecosystems, and being among the least densely populated regions in the world, salmonid populations in this region are rapidly declining as a result of anthropogenic influences with cascading effects on riverine ecosystems^28^. Still, headwater regions across Siberia and northern Mongolia host some of the world’s most pristine rivers and wetlands, receiving some of the highest ecological and chemical status ratings from the European Water Framework Directive standards (e.g., regions including the Selenge River basin headwaters in northern Mongolia^29^). Here, in the remote headwaters of river basins, species including Siberian taimen (*Hucho taimen*), lenok (*Brachymystax lenok* and *B. tumensis*), grayling (*Thymallus spp.*), and pike (*Esox lucius* and *E. reichertii*) are thought to be more abundant compared to other parts of northern Asia^30,31^, as there are fewer disturbances from hatcheries, large habitat modifications to the landscape (e.g., deforestation), and dam development.

The taimen is the world’s largest salmonid, and one of the most ancient extant members of subfamily Salmoninae^32,33^. Reaching up to 2 m in length and 100 kg in weight^34^, taimen reside in home ranges of large and variable size (mean 23 km, maximum up to nearly 100 km^35^) with movements over 200 km recorded for tagged individuals in northern Mongolia (O.P. Jensen, *unpublished data*), a characteristic that could limit the potential for spatial genetic differentiation. However, taimen form pair bonds from weeks to months before moving to spawning areas (unlike other salmonids^36^), and upon reaching an acceptable spawning area, a male will aggressively attack other males within a 20 m radius of the female^37^. Thus, the apparent monogamous nature of taimen spawning behavior, as well as the timing of pair bond formation, could potentially promote spatial genetic differentiation at some scales despite large movements.

Taimen were historically found from the west slope of the Ural Mountains in Eastern Europe to the Pacific Ocean in the east, to the Arctic Circle in the north and the Gobi Desert in southern Mongolia^34^. Similar to most of the world’s largest freshwater fish species^38^, taimen have been negatively affected by human activity and are listed as “Vulnerable” on the IUCN Red List^39^. Anthropogenic disturbances (e.g., overfishing, pollution, mining contamination, energy development) have substantially decreased its native range, including putative extirpations of populations in the Volga, Ural, and Pechora River basins^40^. Further, large stretches of rivers in northern Mongolia have seen local extirpations associated with rapid increases in industrialization^28,40^. Consequently, taimen are listed as threatened or endangered in Mongolia, portions of Russia, Kazakhstan, and China^28^. Though experiencing population declines across much of its historic range, several of the remaining population strongholds exist in the rivers of northern Mongolia and Siberia^31^.

Understanding patterns of genetic diversity and differentiation among river systems and basins at different geographic scales will be critical for informing taimen conservation strategies. Previous research utilized mtDNA and microsatellite markers to illustrate broad phylogeographic relationships in taimen across its native range^41–45^. Variation across three mtDNA regions has shown substantial divergence between populations in different basins, with one distinct clade consisting of the Amur (Pacific) and Lena (Arctic) drainages and the other consisting of the Yenesei (Arctic, specifically referring to the drainage downstream of Lake Baikal) and Khatanga (Arctic) drainages^41^. More recently, Marić et al.^45^ found two distinct haplogroups representing individuals from the (1) Lena and Amur basins and the (2) Volga (Caspian Sea outflow), Ob (Arctic), Yenesei (Arctic), and Khatanga (Arctic) basins. Kaus et al.^46^ used both mitochondrial and nuclear markers and found pronounced population differentiation between populations from the Amur basin (Pacific) and the Upper Yenesei (Arctic) and Selenge (Arctic, specifically referring to the drainage upstream of Lake Baikal) basins with analyses identifying two ancestral genetic clusters that the authors suggested should be considered separate evolutionarily significant units (ESUs). Importantly, none of the aforementioned studies detected any evidence for genetic differentiation or isolation by distance within these large basins, perhaps suggesting that management plans should be implemented at the basin scale. While the lack of evidence for genetic structure across finer geographic scales within basins is consistent with the potential for large movement and population connectivity among river systems, it could alternatively reflect gaps in our understanding and sampling of taimen genetic variation.

Here, we used high throughput sequencing of reduced representation libraries (ddRADseq^47^) to characterize population genetic structure and diversity of taimen within and among several major river drainages in Mongolia and Russia. Specifically, we used single nucleotide polymorphism (SNP) data from multiple sampling sites within one Arctic and two Pacific drainages to (1) characterize levels of genetic differentiation among drainages, (2) evaluate the potential for fine-scale differentiation within drainages, and (3) quantify levels of genetic diversity across sampling sites. We additionally used coalescent-based demographic modeling to infer the demographic and historical context of divergence between two groups of taimen within one Pacific drainage for which we detected unanticipated genetic differentiation at smaller spatial scales. Our results shed further light on the evolutionary history of taimen and suggest the need for more thorough geographic and genomic sampling to facilitate the development of effective strategies for conservation and management.

## Methods

### Sample collection, DNA sequencing, and quality filtering

**Figure 1:**
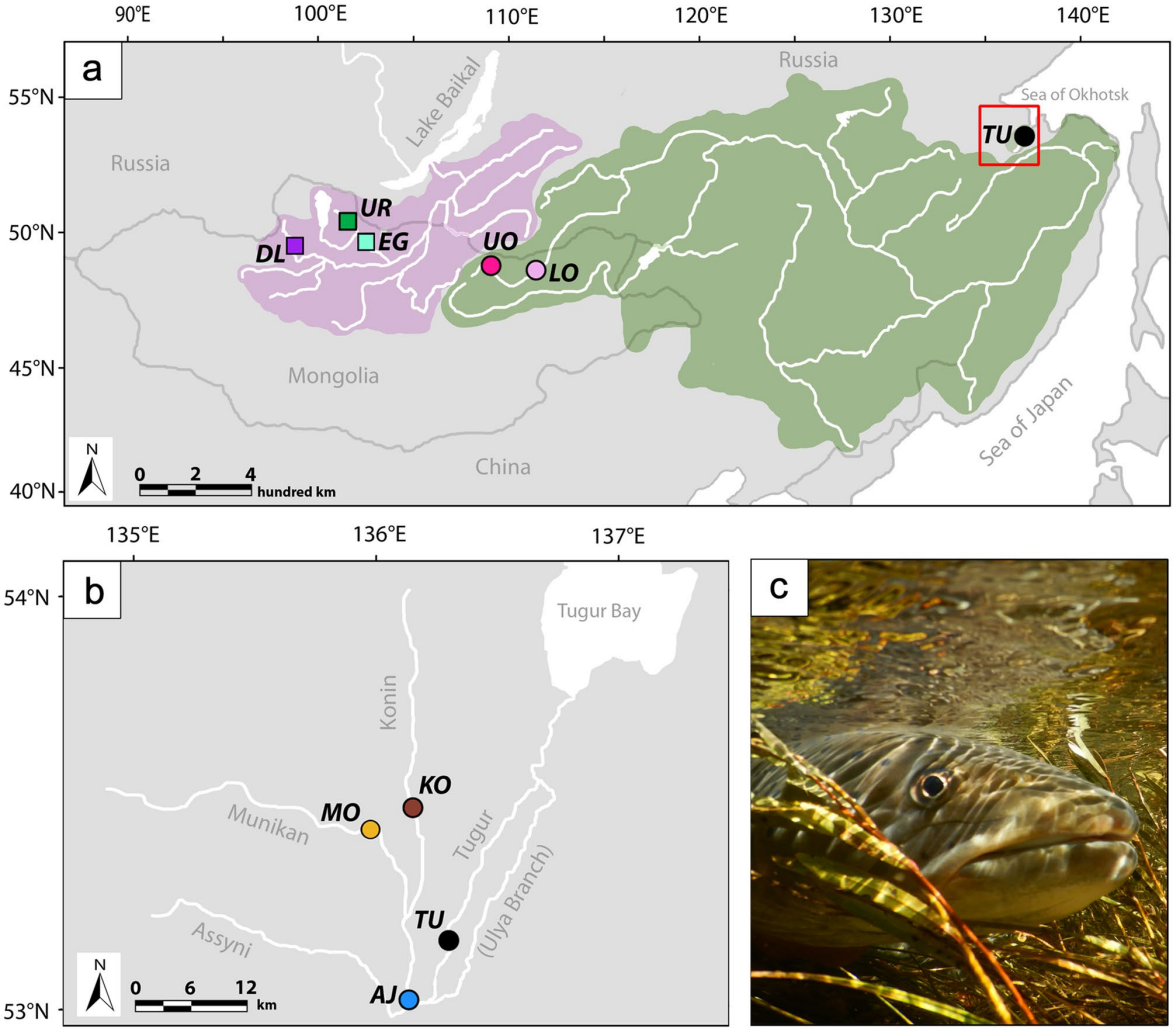
Map illustrating the main drainages from which taimen were sampled from Mongolia and Russia. One Arctic (purple) and two Pacific (green) drainage sampling sites (**a**). The red rectangle denotes the Tugur basin sampling area, shown in greater detail (**b**). Sampling sites correspond to those in Table 1 and include the Delgermörön (DL), Eg (EG), Uur (UR), Lower Onon (LO), Upper Onon (UO), Tugur (TU), Konin/Assyni Junction (AJ), Konin (KO), and Munikan (MU) sampling sites. A recently released taimen rests in the flooded grassland (**c**). Panels (**a**) and (**b**) were modified using Adobe Creative Suite.

We used catch and release fly fishing to obtain samples from 174 taimen using single, barbless hooks, from five sites across northern Mongolia and four sites in southeastern Russia (Fig. 1, Table 1). These sites are distributed across river systems that drain to the Arctic (Eg, Uur, Delgermörön) and those that drain to the Pacific (Amur basin: Upper and Lower Onon; Tugur basin: Konin, Munikan, Konin/Assyni Junction, and Tugur mainstem). Upon capture, small pelvic fin clip samples were taken and the fish were released. Fin clips were stored either in ethanol or dried in coin envelopes for transport. All methods were carried out in accordance with local and national regulations and guidelines (including fishing permits obtained from the local governments), and all experimental protocols were approved by the University of Nevada, Reno, International Animal Care and Use Committee (IACUC protocol ID 20-10-1098). Genomic DNA was isolated from fin clips using Qiagen DNeasy Blood and Tissue kits (Qiagen Inc., Valencia CA), and DNA concentrations were quantified using a Qiagen QIAxpert microfluidics analyzer. Due to variability in DNA yield from fin clip extractions, DNA samples were standardized to within the range of 20–40 ng/uL to ensure similar template concentrations for sequencing library preparation.

**Table 1:** Geographic locations, sample sizes, and capture information for taimen analyzed in this study. Site abbreviations correspond to those in Figs. 1 and 2.

Outflow	Basin	Location name	Latitude, longitude	*N*
Arctic	Selenge	Eg (EG)	49.84, 102.57	13
		Uur (UR)	50.34, 101.87	62
		Delgermörön (DL)	50.11, 98.85	18
Pacific	Amur	Upper Onon (UO)	49.24, 112.02	6
		Lower Onon (LO)	49.12, 111.96	5
	Tugur	Konin (KO)	53.28, 136.26	5
		Munikan (MO)	53.27, 136.12	3
		Konin/Assyni Junction (AJ)	53.07, 136.03	19
		Tugur (TU)	53.11, 136.25	43

We used a reduced representation approach based on two restriction endonucleases to generate ddRADseq libraries. DNA was digested with restriction endonucleases *Mse*I (4-base recognition site) and *Eco*RI (6-base recognition site). To the *Eco*RI cut-sites, we ligated Illumina adaptors embedded with unique 8–10 bp barcode sequences which were used to tag DNA from each individual. An Illumina sequencing adaptor was ligated to the *Mse*I cut-sites. We then pooled the uniquely barcoded samples and amplified fragments using Illumina PCR primers. To reduce the portion of the genome sampled for sequencing, libraries were size selected for fragments ranging from 350 to 450 bp using a Pippin Prep unit (Sage Science, Beverly, MA) at the University of Texas Genome and Sequencing Analysis Center (Austin, TX). Full details on the laboratory methods used for library preparation can be found in Ref.^48^ Size selected libraries were then sequenced on two lanes of an Illumina HiSeq 2500 at the University of Wisconsin-Madison Biotechnology Center’s Genome Center (Madison, WI).

Raw sequencing data was filtered for contaminant sequences including *E. coli* and *PhiX*, and for Illumina sequencing adaptors, using bowtie2_db^49^ and a pipeline of Perl and bash scripts (http://github.com/ncgr/tapioca). Importantly, barcode sequences all differed by a minimum of three bases, allowing us to detect one or two base sequencing errors within them. A custom Perl script (available at DRYAD doi: https://doi.org/10.5061/dryad.wstqjq2kd) was used to correct 1 or 2 bp sequencing errors in barcode sequences, remove barcodes and cut-site associated bases, and match sequences with individual sample names. Reads were then split into fastq files specific to each individual. We then removed individuals represented by volumes of sequencing data lower than the 1st quartile of the distribution spanning all samples to reduce the fraction of missing data and to increase the number of loci retained for analyses of a sufficient number of samples per sampled locality.

### Alignment, variant calling, and filtering

Since we quantified substantial genetic differentiation among samples from the two major outflows (Pacific and Arctic, see “Results” section below), we conducted separate analyses based on: (1) all individuals (full dataset); and (2) a subset of individuals sampled from populations in the Pacific drainages (Pacific subset). We did not analyze data separately for the Arctic subset, as analysis of the full dataset (in “Results” section) suggested limited genetic structure among populations within this drainage. All methods for alignment, variant calling, and filtering were identical for both datasets, except for the de novo reference generation, which was implemented separately with the different sets of samples. As there were no reference genomes available for any closely related taxa, we used a de novo clustering approach to build a reference of genomic regions sampled with our sequencing approach as a basis for aligning all of the reads. We used CD-HIT^50^ to generate contig consensus sequences (partial reference hereafter) built from clustering the unique sequences in our entire dataset with a minimum match percentage of 90%. This de novo clustering algorithm, also utilized by the commonly used RADseq pipeline dDocent^51^ was used to generate a partial reference to serve as a target for subsequent read mapping. We used bwa v0.7.5^52^ to map sequences generated for each individual to the partial reference based on an edit distance of three. Sequence alignment map (.sam) files were converted to binary alignment map (.bam) files with samtools v1.3^53^, before samtools v1.3 and bcftools v1.3^53^ were used to identify and call variants across the alignments of all individuals. We calculated genotype likelihoods for SNPs at sites with a minimum base quality of 20, maximum coverage depth of 100, minimum map quality of 20, minimum site quality of 20, and minimum genotype quality of 10. We used vcftools v0.1.14^54^ to further filter called variants. We retained only bi-allelic SNPs with minor allele frequencies (MAF) greater than 0.04, and those where at least 60% of individuals had at least one read. We randomly sampled one SNP per 100 bp contig and discarded individuals with missing data at more than 30% of loci.

As mis-assembly of reads representing paralogous regions can lead to genotyping error in high throughput sequencing data^55^, we took several steps to mitigate the potential influence of such loci. First, we used vcftools v0.1.14 to remove loci with exceptionally high coverage depth per individual, greater than or equal to 50. We then additionally identified and removed potentially paralogous loci using the HDplot approach described in Ref.^55^. This method identifies potential paralogs in sequence data from populations based on deviations from the expected frequency of heterozygotes and from the expected 1:1 ratio of read counts for alternate alleles in heterozygotes. Here, we retained loci with heterozygosity (*H*) levels between 0 and 0.6, and read ratio deviance (*D*) between − 18 and 18. We took these steps to exclude potentially misassembled genomic regions representing duplicate or diverged duplicate loci.

### Population genetic analyses

We quantified population structure without a priori sample information using the Bayesian ancestry-based model entropy v1.2^56,57^ which is based on the correlated allele frequency model of structure^58^. We used entropy to infer the number of *k* ancestral populations represented by the data and to estimate admixture proportions (*q*) for individuals. Importantly, this model accounts for statistical uncertainty arising from sequencing and alignment error and stochastic variation in coverage depth inherent in low to medium coverage sequencing data^59,60^. Because entropy provides posterior estimates of genotype probabilities at each locus for each individual, it allows for the incorporation of genotype uncertainty into downstream population genetic analyses. To seed and speed the convergence of Markov chain Monte Carlo runs, we first generated starting values for the *q* parameter. We conducted a principal component analysis (PCA) on the covariance matrix of genotype likelihoods calculated above using the prcomp function in R version 4.1^61^ and then used *k*-means clustering and linear discriminant analyses (LDA) to estimate ancestry proportions for each individual for models representing *k* = 2 through *k* = 9 (or through *k* = 6 for the Pacific dataset). We ran entropy models for *k* = 2 through *k* = 9 (or *k* = 6) ancestral groups, with 5 replicate chains per *k*. We ran 100,000 MCMC iterations, retaining every tenth step after a burn-in of 30,000 steps. Model fit was assessed using the deviance information criterion (DIC), where lower DIC values represent better model fit. We conducted entropy runs separately for (1) the set of SNP genotype likelihoods for all individuals and (2) the subset of samples representing locations within the Pacific drainages. We used genotype probabilities from entropy for the majority of the population genetic analyses described below.

We additionally characterized genetic variation with PCA using the prcomp function in R. As metrics of pairwise genetic differentiation among populations, we calculated Hudson’s F_ST_^62^ and Nei’s D^63^ based on population allele frequencies. As metrics of genetic diversity for each sampling location, we calculated nucleotide diversity (*θ*_*π*_, or the average number of pairwise differences between sequences^64^), Watterson’s theta (*θ*_W_, or the number of segregating sites^65^), and the scaled difference between the two (Tajima’s *D*^66^) using methods that incorporate genotype uncertainty implemented in ANGSD^67,68^. We used the de novo artificial reference genome and individual .bam files to estimate site allele frequency likelihoods using the “doSaf 1” tool. We then used site allele frequency likelihoods as input for REALSFS^68^ to generate folded site frequency spectrum likelihoods. Using “doSaf 1,” we calculated posterior allele frequency probabilities. Lastly, we used the thetastat utility from ANGSD to estimate per-locus measures of each diversity metric and generated the per-population average of these values over all contigs and nucleotides. For comparison with other studies, we also calculated expected heterozygosity based on allele frequencies.

As we found unexpected evidence for divergence between taimen in the Tugur mainstem and its tributaries, we used a coalescent-based demographic modeling approach to explore parameters characterizing the divergence and demographic history of these two populations. The site frequency spectrum (SFS) contains the signatures of divergence and demographic processes (e.g., time, migration, changes in effective population size), and high throughput sequencing data has substantially improved our ability to estimate such parameters from population genetic data^69,70^. Before generating the SFS, we further filtered the vcf file generated above. First, we removed variants with MAF < 0.1 to guard against rare variants that could represent sequencing errors and trimmed outlier loci with F_ST_ > 0.15 (the 0.95 quantile of the F_ST_ distribution) between the two populations. We generated the unfolded SFS for each population using easySFS (https://github.com/isaacovercast/easySFS#easysfs) on the stringently filtered .vcf file, down sampling populations to sizes of 10 and 10 (–proj 10, 10).

Using the unfolded SFS, we estimated demographic parameters for eight different models using coalescent simulation and a maximum likelihood framework in fastsimcoal2^69^. These models represented two-population divergence (Tugur mainstem and Tugur tributaries), with and without migration, and with and without population expansion or contraction. We ran 50,000 coalescent simulations per replicate and a total of 50 replicates, with minimum (-n) of 100,000 simulations for a total (-L) of 40 cycles. We used a mutation rate for salmonids (Salmoninae) of 8e−9 bp per generation for model estimation (coho salmon^71^), and to estimate coalescent effective population size. For each model, the replicate with the smallest difference between the maximum expected likelihood (MEL) and the maximum observed likelihood (MOL) represented the best-fit run^67^. To account for differences in the number of parameters included in each model, we calculated AIC scores for each model’s best-fit run. We then calculated ∆AIC to compare models.

Following parameter estimation for the best fit model, we calculated 95% confidence intervals for each parameter. Using the maximum likelihood parameters from the *_maxL.par file, we generated 100 bootstrap replicates of the SFS. Next, we estimated parameters of these 100 SFS using the same 50-replicate analyses described above. The best-fit model parameter estimates for each of the new 100 simulated SFS were used to calculate mean parameter estimates and subsequently to infer 95% confidence intervals.

## Results

### Full dataset

After filtering for contaminants and removing individuals lacking sufficient sequencing data, we retained 174 individuals with a mean number of 1,898,867 reads per individual. bwa mapped reads from all individuals onto the de novo partial reference consisting of 221,178 genomic regions. After variant calling and filtering based on sequencing coverage and quality, we retained 7597 loci with MAF > 0.04. We additionally discarded 1551 SNPs that potentially represented paralogous regions, leaving a final set of 6046 SNPs from 174 individuals (mean coverage = 10.1X per locus per individual). Sequence data is available on NCBI’s Short Read Archive (accession PRJNA745962; https://dataview.ncbi.nlm.nih.gov/object/ PRJNA745962). Both the sequence data and the vcf file are available at DRYAD (doi: https://doi.org/10.5061/dryad.wstqjq2kd).

**Figure 2:**
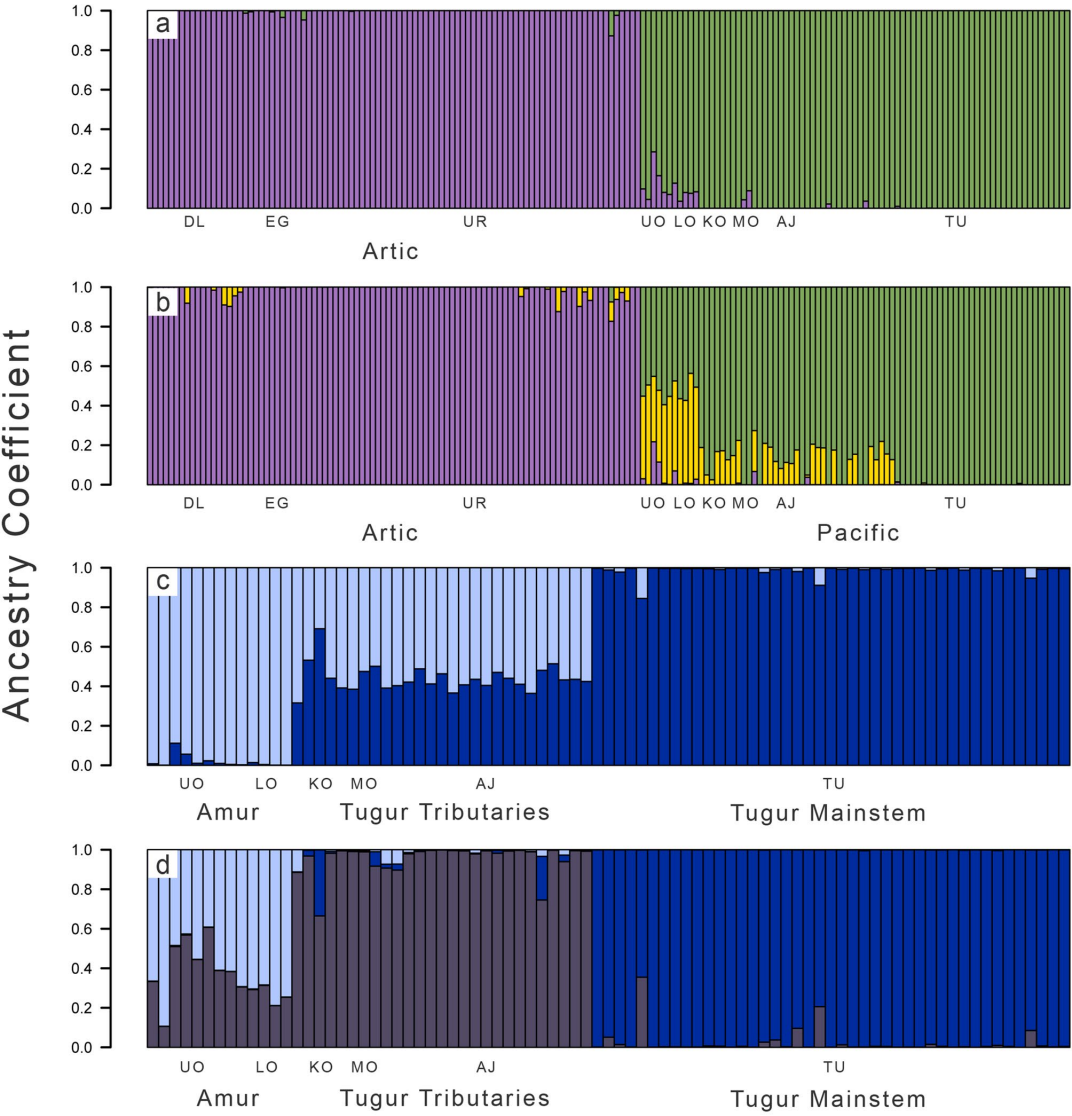
Ancestry coefficient estimates (*q*) generated with entropy for analyses based on all sampled individuals (**a**, **b**) and for separate analyses based on the subset of individuals from the two Pacific drainages (**c**, **d**). Vertical bars represent individuals, and colors correspond to the admixture proportions for each of *k* clusters. For both sets of analyses, the *k* = 2 models (**a**, **c**) fit the data best. Models for *k* = 3 (**b**, **d**) are additionally shown for each set of analyses as the revealed patterns of clustering further illustrate population genetic structure within river systems. As in Fig. 1, sampling sites correspond to those in Table 1 and include the Delgermörön (DL), Eg (EG), Uur (UR), Lower Onon (LO), Upper Onon (UO), Tugur (TU), Konin/Assyni Junction (AJ), Konin (KO), and Munikan (MO) sampling sites. Plotting was completed using R software.

**Figure 3:**
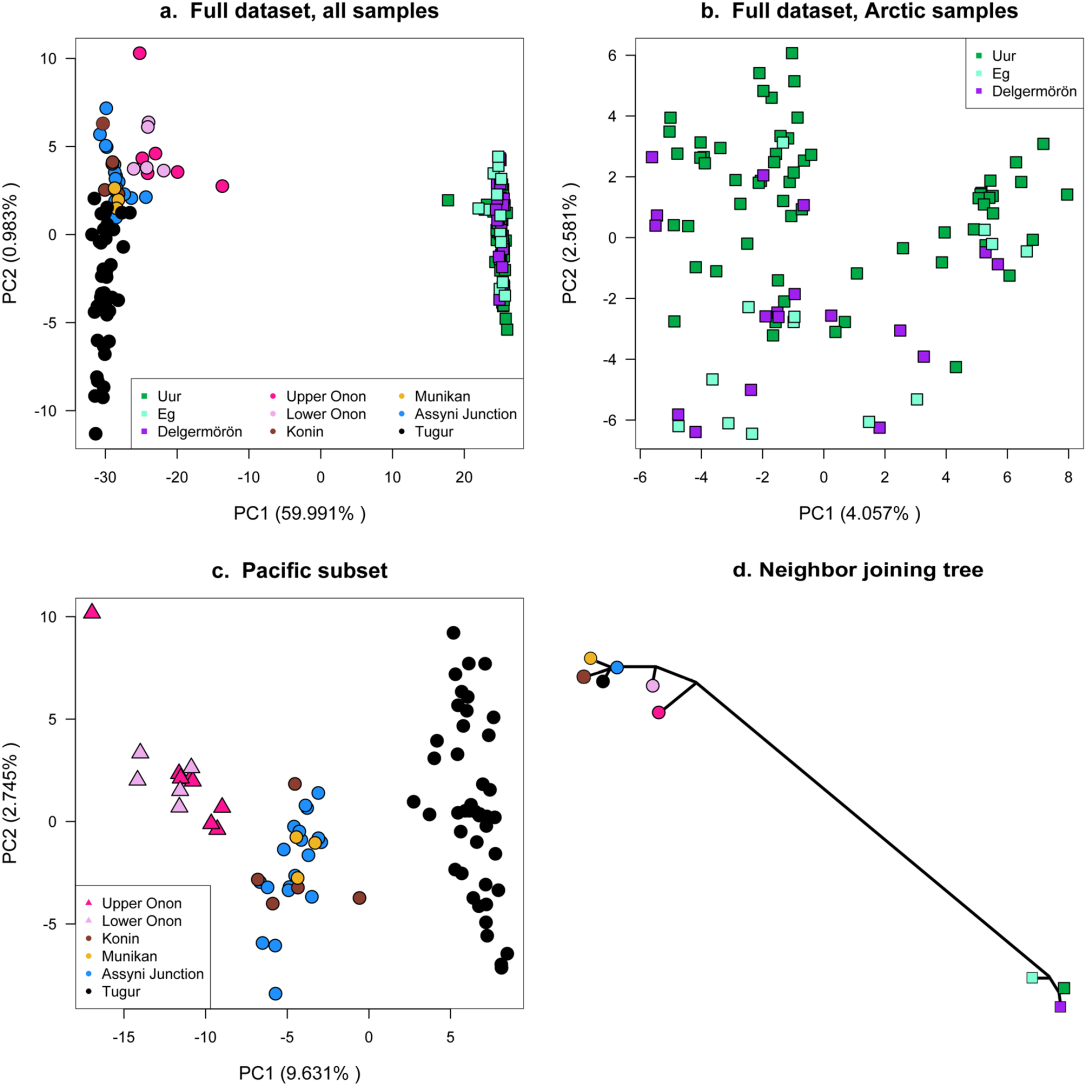
Genetic variation among taimen illustrated with PCA (calculated using R software) of 6046 SNPs called in all sampled individuals (**a**), Arctic individuals (**b**), and in 3961 SNPs called separately for individuals from the Pacific drainages (**c**). In panel (**a**), samples from the Pacific drainages are represented by circles, and those from the Arctic drainage by squares. In panel (**c**), samples from the Amur basin are represented by triangles while those from the Tugur basin are represented by circles. The neighbor joining tree in panel (**d**) supports the deep divergence between Arctic and Pacific drainages, where symbols represent populations shown in panel (**a**).

Pronounced genetic differentiation was evident between taimen sampled from the Arctic and Pacific drainages across all analyses (Figs. 2a, 3). DIC values from entropy indicated that the *k* = 2 model best fit the data, though the *k* = 3 model had similar support and illustrated additional population differentiation (Supplementary Table S1). Individuals were assigned nearly 100% to one of two ancestries (Fig. 2a): the Arctic with the single Selenge drainage (Eg, Uur, and Delgermörön Rivers), and the Pacific with the Amur (Upper and Lower Onon River sites) and the Tugur (Konin, Munikan, Konin/Assyni Junction, and Tugur Rivers) drainages (Fig. 1). The *k* = 3 model reflected the same pattern for the Arctic versus Pacific drainages, but individuals were assigned with variable ancestry across several Pacific sites exhibiting differentiation (Fig. 2b).

PCA of the genotype probabilities revealed genetic structure similar to ancestry estimates from entropy (Fig. 3a). PC 1 explained 59.99% of variation in the data, separating individuals and populations from the Arctic and Pacific drainages. PC 2 explained only 0.98% of variation in the data, but suggested finer-scale structure within the Pacific drainages. Pairwise measures of genetic divergence among sites from the two outflows (Arctic and Pacific) were high (F_ST_ range = 0.209–0.258, Nei’s D range = 0.1405–0.1823; Supplementary Table S2) consistent with independent evolutionary histories and substantial isolation of drainages (Table 2). A neighbor joining tree based on pairwise estimates of Nei’s D among sampling localities provided and alternative visualization of hierarchical genetic structure consistent with all other population genetic analyses (Fig. 3d).

**Table 2:** Population genetic diversity for the full set of 174 individuals, including estimates based on population allele frequencies (*H*_*E*_) and those based directly on DNA sequence variation across the sampled genomic regions (the average number of pairwise differences between sequences, or *θ*_π_, the number of segregating sites, or *θ*_W_, and the scaled difference between these two, or Tajima's *D*).

Outflow	Basin	Site	*H*_*E*_	*θ*_*π*_	*θ*_*π*_ low	*θ*_*π*_ high	*θ*_*W*_	*θ*_*W*_ low	*θ*_*W*_ high	*D*	*D* low	*D* high
Arctic	Selenge	DL	0.118	0.001187	0.001177	0.001197	0.000774	0.000768	0.000779	− 0.00665	− 0.00668	− 0.00662
		EG	0.121	0.001348	0.001338	0.001358	0.001006	0.000100	0.001013	− 0.01024	− 0.01026	− 0.01021
		UR	0.120	0.001377	0.001367	0.001387	0.000999	0.000993	0.001004	− 0.01266	− 0.01270	− 0.01263
Pacific	Amur	UO	0.227	0.002090	0.002077	0.002103	0.001777	0.001767	0.001787	0.00210	0.00207	0.00213
		LO	0.217	0.001757	0.001744	0.001770	0.001506	0.001496	0.001516	0.00475	0.00472	0.00478
	Tugur	KO	0.214	0.001646	0.001635	0.001658	0.001391	0.001382	0.001400	− 0.00025	− 0.00028	− 0.00022
		MO	0.213	0.001875	0.001861	0.001889	0.001672	0.001661	0.001684	0.00835	0.00832	0.00838
		AJ	0.221	0.001775	0.001765	0.001786	0.001585	0.001578	0.001591	− 0.02457	− 0.02460	− 0.02454
		TU	0.230	0.001694	0.001682	0.001705	0.001186	0.001179	0.001192	− 0.00942	− 0.00946	− 0.00938

High and low columns represent upper and lower 95% confidence intervals for each metric.

Genetic diversity varied across the sampled geographic regions. Mean genetic diversity across all nine sampling sites was 0.00164 for *θ*_*π*_ (range 0.00119–0.00209) and 0.00132 for *θ*_W_ (range 0.00077–0.00178). Genetic diversity was consistently higher in Pacific drainages (mean *θ*_*π*_ = 0.00181, range 0.00165–0.00209; mean *θ*_W_ = 0.00152, range 0.00119–0.00178) than in the Arctic drainage (mean *θ*_*π*_ = 0.00130, range = 0.00119–0.00138; mean *θ*_W_ = 0.00093, range = 0.00077–0.00101), as was *H*_*E*_ (Arctic mean = 0.1195, range = 0.118–0.121; Pacific mean = 0.2203, range = 0.213–0.230). No populations from the Arctic drainage had confidence intervals that overlapped with those in the Pacific drainages. Importantly, for taimen localities sampled in the current work, sample size was unrelated to both genetic diversity metrics (sample size vs. heterozygosity: *r* = − 0.327, *P*—0.323; sample size vs. nucleotide diversity: *r* = − 0.424, *P* = 0.253; Supplementary Fig. S1).

### Analyses of the Pacific drainages

Given pronounced genetic differentiation between the Arctic and Pacific drainages and evidence for finer-scale differentiation among river systems within the Pacific drainages, we conducted assembly and variant calling separately for Pacific populations. Unique reads were assembled into a partial reference consisting of 160,858 contigs. Following reference mapping, variant calling, subsequent bioinformatic processing, and paralog filtering, we retained 3961 SNPs from 83 individuals (mean coverage = 10.4X per locus per individual) for the Pacific subset. The vcf file associated with the Pacific subset is available at DRYAD (doi: https://doi.org/10.5061/dryad.wstqjq2kd).

Although pairwise measures of genetic differentiation among sampling sites across this drainage were relatively low (Supplementary Table S2), analyses illustrated finer-scale patterns of differentiation that were less evident in analyses of the full data set. The *k* = 2 entropy model fit the data best, although the *k* = 3 model had similar DIC support (Supplementary Table S1), and further illustrated population structure. Across analyses, clear genetic differentiation was evident between the Amur (Upper and Lower Onon sites) and Tugur drainages (Figs. 2c, d, 3b). Both the *k* = 2 and *k* = 3 eentropy models assigned individuals from the Amur and Tugur drainages to different ancestral clusters (Fig. 2c, d), and PCA clearly separated individuals from the two drainages (Fig. 3c).

There was unexpected genetic differentiation among sampling sites within the Tugur basin; taimen from the headwater tributaries (KO, MO, AJ; n = 37) were differentiated from samples taken in the mainstem Tugur (TU; n = 43; Fig. 1b). Ancestry was assigned differentially to separate clusters in both the *k* = 2 and *k* = 3 entropymodels (Fig. 2c, d), and the tributary populations formed a non-overlapping cluster in PC space intermediate between samples from the Tugur and the Amur drainages (Fig. 3c). Although overall genetic differentiation was subtle (F_ST_ range = 0.012–0.029; Supplementary Table S2), these analyses nonetheless indicate the presence of finer-scale differentiation among sampling sites in the Tugur than in the other drainages we examined.

**Figure 4:**
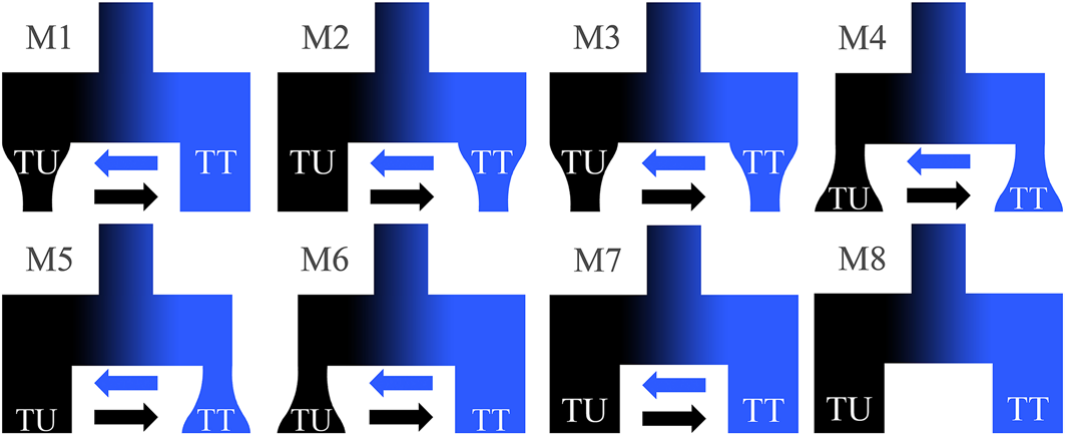
Representations of each two-population model tested with fastsimcoal2, where TU represents the mainstem population, TT represents the tributaries population, and arrows represent gene flow. Model numbers and parameters correspond to those listed in Table 3.

**Figure 5:**
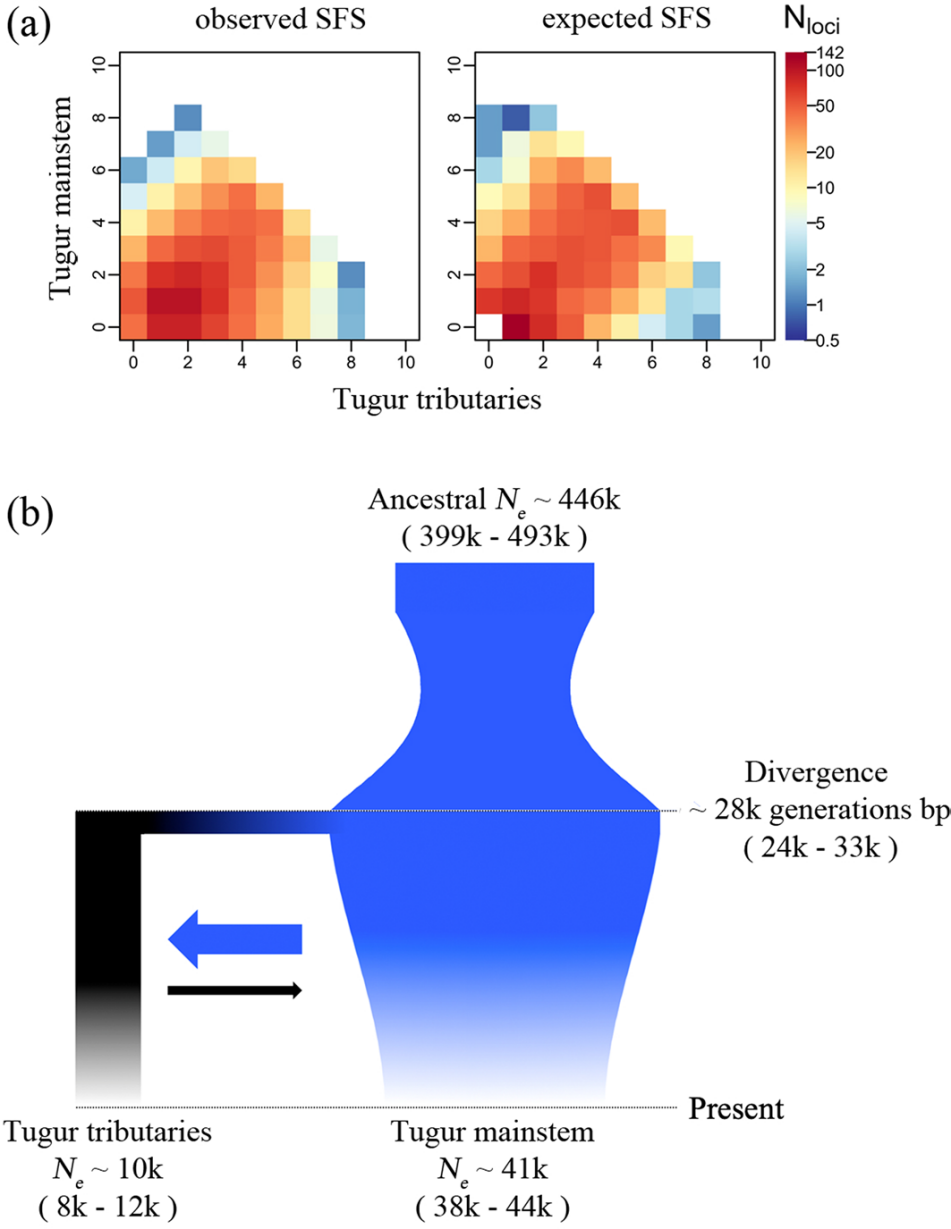
The expected versus observed SFS for the best fit model shows substantial overlap (**a**). Panel (**b**) shows the schematic representing the best fit model (model 1) from demographic inference using fastsimcoal2. Coalescent *Ne* is given for population, with branch and arrow width corresponding to population size and level of gene flow, respectively (but not drawn to scale). Numbers in parentheses represent 95% confidence intervals.

After the more stringent filtering with vcftools v0.1.14, we retained 1971 variants from which we constructed the unfolded SFS. Coalescent simulations using fastsimcoal2 were run for eight models spanning variation in divergence and demography of the Tugur mainstem and Tugur tributary populations (see Table 3 for parameter estimates and model comparison metrics, and Fig. 4 for model schematics). Importantly, all models including migration had substantially better fits than the model without migration. Model likelihoods were similar across models including migration, but the best fit model included initial divergence, after which coalescent *N*_*e*_ remained constant in the tributary population but contracted in the mainstem population (Table 3; Fig. 5). Parameter estimates indicated divergence at ≈ 28 k generations ago based on a mutation rate of 8e−9 (mean ≈ 393 kya based on mean generation time of 13.8 years [range ≈ 195–594 kya based on generation time range 6.9–20.8 years (mean ± 2 standard deviations)]^34,72^; Fig. 5). Generation time was calculated from age-frequency data from the Tugur River (M.R. Sloat, *unpublished data*) and fecundity-at-age data presented by Ref.^34^. Following the ancestral divergence event, asymmetrical gene flow was inferred between mainstem and tributary clusters, with greater gene flow from the mainstem to tributaries. Coalescent *N*_*e*_ for tributaries was substantially lower than that of the mainstem (≈ 10,000 vs. ≈ 41,000), and the model indicated population contraction in the latter.

**Table 3:** Demographic modeling parameter estimates for each of eight 2-population models.

Model description	Model #	Growth parameters		*N*_*e*_		Ancestral *N*_*e*_	T_divergence_ (generations)	Migration		ΔModel likelihood	ΔAIC
		TU	TT	TU	TT			TU → TT	TT → TU		
2 Population with gene flow	1	−	0	40,999	10,058	446,164	28,480	0.00356	0.00435	31.13	−
				(38,052–43,946)	(7998–12,119)	(398,862–493,466)	(23,685–33,276)	(0.00279–0.00434)	(0.00386–0.00485)		
	2	0	−	3302	83,980	734,783	5046	0.00251	0.59000	39.68	39.36
	3	−	−	47,370	81,411	253,462	53,643	0.00377	0.00027	36.32	23.88
	4	+	+	21,677	72,582	380,356	2587	0.00702	0.00574	35.73	21.17
	5	0	+	505	54,433	743,812	2274	0.00489	0.00605	38.05	31.84
	6	+	0	18,046	7769	816,466	5739	0.00170	0.00401	36.72	25.73
	7	0	0	687	1724	523,521	1247	0.00596	0.00464	35.67	20.88
2 Population	8	0	0	435	791	110,201	38	−	−	136.83	484.7

TU refers to Tugur mainstem, while TT refers to Tugur tributaries.

Growth parameters for each population represent expansion (+), contraction (−), and no change (0) following the ancestral divergence.

Model numbers correspond to those in Fig. 4

For the best fit model (model 1), 95% confidence intervals are given in parentheses.

## Discussion

Using high throughput sequencing of reduced representation libraries, we documented hierarchical patterns of genetic differentiation among taimen sampled across multiple drainages. We found pronounced divergence among taimen from the Arctic and Pacific drainages, but also recovered more subtle patterns of differentiation within and among river systems that drain to the Pacific. Although our spatial sampling of the distribution was limited, our analyses indicate the potential for genetic differentiation at finer scales within basins and even within specific river systems. This aspect of our results differs from several past studies and was potentially influenced by different spatial and more thorough genomic sampling. Below, we discuss our results in the context of past genetic work on taimen, with consideration of how current and future population genetic analyses could inform their management and conservation.

### Deep genetic divergence between Arctic and Pacific drainages

Our population genetic analyses illustrate substantial genetic differentiation consistent with significant historical isolation between taimen from the Arctic and Pacific drainages (mean F_ST_ = 0.24; Fig. 1, Supplementary Table S2), similar to past studies based on smaller sets of genetic markers^41,45,46^. Additionally, maximum likelihood phylogenetic analyses (RAxML^73^) based on 1229 SNPs in a concatenated alignment similarly illustrated deep divergence among taimen sampled from the Pacific and Arctic drainages (Supplementary Methods, Supplementary Fig. S2). Genetic differentiation across systems would be expected given the geologic barriers separating these drainages. Though the Amur and Selenge basins are separated by only a few kilometers (Fig. 1), the Khentii Mountains arising from the Baikal Rift system (last active during the Pliocene^74^) form a continental divide that likely severed any recent connectivity between the Arctic and Pacific river systems. This geologic feature is associated with deep phylogeographic breaks for other taxa occurring in this region, including other salmonids (*Thymallus*^75,76^, *Brachymystax*^46,77^), as well as other groups of freshwater fishes (*Cottus*^78^, *Esox*^79^). Divergence time estimates from mtDNA analyses indicate divergence across this divide in the range of 1–2.5 mya for *Cottus*^78^ and 0.5–2 mya for taimen^41,45^.

Taimen from the Arctic and Pacific outflows do differ in a few key morphological traits, including average mass per length, and density and size of spot patterns (Mikhail Skopets and M.R. Sloat, *unpublished data.*). This variation is consistent with isolation and independent evolution, and could possibly be due in part to ecological differences among drainages on either side of this divide, such as riverscape and landscape topography^35,72^, constituent riparian species composition^35^, and marine food web subsidies from seasonal spawning runs of chum salmon in the Tugur basin, but not the Selenge basin^72^. Unfortunately, the geographically sparse and clumped nature of our sampling led to a strong correlation between geographic and environmental distances (based on 19 BioClim variables) among sampling localities, which precluded formal tests of environmental influences on spatial genetic structure.

We did not detect genetic differentiation among sites within the Arctic drainage (Figs. 2, 3), though only three locations, all within the Selenge basin, were sampled in this drainage. Future studies could benefit from additional sampling across the Arctic drainage, particularly upstream and downstream of Lake Baikal, which may have presented a migration barrier to the predominantly riverine taimen. This apparent absence of genetic structure is consistent with past studies based on microsatellite and mtDNA data^45,46^, and with the possibility of population connectivity among sites separated by hundreds of kilometers. Limited genetic differentiation across broad spatial scales would not be surprising given the large size, long lifespan, and substantial opportunity for movement in taimen^35^. Moreover, the region’s glacial history suggests that populations in the Arctic are likely younger than those in the Pacific drainages. Northwestern Siberia experienced repeated Pleistocene glaciations which blocked north-flowing rivers and formed ice dam lakes^80–82^, whereas the more southern Pacific drainages are thought to have been less influenced by glaciation^83^ (but see Ref.^80^). A younger taimen lineage in the Arctic drainage would be consistent with limited differentiation among populations often seen in Arctic fishes^84,85^, and also with variation in genetic diversity among the regions we sampled.

For all metrics, levels of genetic diversity were consistently lower for the three Arctic drainage sites than those from Pacific drainage sites (Table 2). Measures of nucleotide diversity were relatively low, but well within the range of published nucleotide diversity estimates across salmonids^86–89^. For example, our values for taimen were similar to those reported for Atlantic salmon (*Salmo salar*, overall nucleotide diversity of 3.99e−4; Ref.^90^), a species that has also been strongly influenced by recent glacial periods^90^. Lower diversity in the Arctic drainage is consistent with the hypothesis that Arctic populations are younger, and/or suffered recent bottlenecks during Pleistocene glacial activity in this region^82^.

### Fine-scale genetic structure within the Pacific drainages

Our analyses revealed clear, though less pronounced, genetic differentiation between the two Pacific drainages (Amur and Tugur). Taimen from the Onon River (Amur basin) were differentiated from those occurring 1800–2000 km to the east in the Tugur basin (mean F_ST_ = 0.033). This pattern could be consistent with the Yablonovy and Stanovoi Mountain ranges acting as a divide for aquatic fauna separating the headwaters of the Lena (just north of the Tugur) and Amur basins^91^, although a lack of sampling over a large area between the Onon and Tugur sites limits our understanding of geographic features associated with spatial genetic structure. As in the Arctic drainage, we did not detect evidence for differentiation among sampling sites within the Amur basin. However, given the size and complexity of the Amur basin combined with the population structure documented across the basin in other salmonids (e.g., *Thymallus*^76^), it is possible that more thorough sampling could reveal additional structure.

Our analyses revealed unexpected evidence for fine-scale genetic differentiation between two groups of taimen sampled in the Tugur basin. Though sampling locations within this basin are highly proximate (often separated by less than 10 km, Fig. 1) and well within average home ranges of taimen^35,92^, we observed distinct genetic differentiation between the Tugur River mainstem and its tributaries (Figs. 1b, 2c, d, 3c). Differentiation among these groups was subtle but clear (Supplementary Table S2), as individuals from the mainstem and tributary groups were completely identifiable and formed non-overlapping groups in PCA and ancestry-based analyses (Figs. 2d, 3c). No evident barriers to movement (and thus spawning) have been observed in this region, and taimen from these sites are not known to differ morphologically. Nonetheless, the pattern of consistent differentiation among these sites indicates that some barrier to gene flow likely exists. Ecological variation among tributaries and the mainstem has been noted, including differences in water flow, water level, and chemical composition, as well as the timing of food availability (e.g., variation in chum salmon spawning^93^), suggesting ecological factors may underlie isolation. Reproductive isolation has evolved within a number of salmonid species due to spatiotemporal differences in spawning (spring vs. fall Chinook salmon^26^), or morphological specialization (dwarf and normal *Coregonus clupeaformis*^94^; benthic and limnetic *Salvinus alpinus*^95^; pelagic and littoral feeding *Thymallus nigrescens*^96^). Additionally, although taimen populations typically do not differentiate at smaller spatial scales, anadromous Sakhalin taimen (*Parahucho perryi*) demonstrate population genetic structure arising from differences in spawning grounds and homing behavior^97^. While we are unaware of such mechanisms underlying divergence in taimen, our results indicate the potential for differentiation at smaller geographic scales than previously detected and the need for further study to understand its evolutionary causes and consequences for management.

Given the unexpected differentiation within the Tugur basin, we compared empirical and simulated SFS for models representing different divergence scenarios to consider the timing and demographic context of this divergence. All seven of the models incorporating migration had strongly improved fit compared to the model without migration (Table 3), consistent with a history of allopatric divergence followed by secondary contact and gene flow. The best fit model included bidirectional gene flow, with population size constant in the Tugur tributaries and contracting in the mainstem. The divergence time estimate for this model of ~ 28 k generations would correspond to divergence at 195–594 kya, depending on generation time estimates. The estimated coalescent *N*_*e*_ was substantially larger in the Tugur mainstem than the tributaries, while migration probabilities were higher from the tributaries into the mainstem. It is worth noting that parameter estimates for these models can be affected by mutation rate, changes in migration and population size over time, and bioinformatic processing of sequence data^98,99^, and also that the additional models including migration had similar likelihoods to the best fit model yet very different parameter estimates (Table 3). Importantly, coalescent *N*_*e*_ estimates can be heavily affected by changes in these modeling input values, as seen in our substantially higher coalescent *N*_*e*_ for the best fit model than empirical *N*_*c*_ estimates for taimen^72^. Denser data and sampling would improve our ability to evaluate such models and characterize this history.

A possible, if not likely, scenario underlying divergence in the Tugur basin is that ancestral tributary taimen diverged in isolation in the eastern Amur, before paleohydrological connections allowed colonization of the Tugur basin. Consistent with this hypothesis, taimen in the Tugur tributaries are intermediate with respect to those from the Tugur mainstem and the western Amur basin samples in both PCA and ancestry-based analyses (Figs. 2, 3). Although the Tugur and Amur basins have no contemporary hydrologic connectivity and the hydrologic history of this region is not well understood, faunal similarities are consistent with paleohydrological connections among these drainages during the Pleistocene. For example, mtDNA haplotype sharing among the Amur and Tugur basins has been documented in blunt-nosed lenok (*Brachymystax lenok*), suggesting a history of such connectivity^77^. The location of a potential paleo connection between the Tugur and Nimelen, a lower Amur tributary, is apparent in a low-relief area where active channels in tributaries of the two rivers are separated by < 1 km and by a drainage divide of < 10 m. One hypothesized scenario is that the Tugur was forced south and joined the lower Amur during Pleistocene “back-arc glaciation” of a Sea of Okhotsk marine ice sheet and the Stanovoi glacier complex to the west before drainages reorganized and became independent during glacier retreat^80,100^. A similar pattern of glaciation has been hypothesized to generate paleohydrological connections between the proto Tugur drainage and mid-Amur drainages of the Bureya and Zeya Rivers (see Fig. 6 of Ref.^100^).

Understanding the historical and geographic context of differentiation in the Tugur basin as well as the mechanisms potentially maintaining it will require further study. Our results are based on relatively sparse spatial sampling only in the western Amur basin. More thorough sampling of the eastern Amur basin, especially where it nears the Tugur, will be necessary to more thoroughly characterize the geographical context and origin of divergence among taimen in the Tugur basin. Similarly, additional sampling within the Tugur basin could improve understanding of the spatial distribution of the genetically differentiated groups detected here. As importantly, an understanding of ecological, morphological, and life history variation among taimen in this system will be critical for understanding potential mechanisms underlying and maintaining differentiation. Further work here is warranted as the Tugur River continues to support the largest individuals of the world’s largest salmonid^101^, and the majority of the watershed is protected within the Tugursky Nature Reserve, a regional zakaznik (equivalent to an IUCN Category IV protected area).

### Implications for conservation and management

Given substantial and widespread declines of taimen populations, an understanding of genetic diversity and spatial genetic structure could be critical for informing taimen conservation. The demarcation of taimen management units could become important as anthropogenic influences known to impact fish populations (e.g., see Refs.^102,103^) increase in frequency and intensity, and as translocation efforts are considered for regions where populations have declined or have been extirpated. Genetic diversity is often considered a key parameter for conservation and restoration, as it is commonly viewed as a proxy for population resilience and evolutionary potential^104,105^. Nucleotide diversity estimates for all of our sampling sites were in the range of estimates published for other salmonids^86–89^, and do not reflect severely reduced diversity. Although, Tajima’s *D* estimates were slightly positive for the Amur basin, which could be consistent with population declines here. However, our sampling was limited to regions with healthy river systems and those that have yet to exhibit strong population declines, and may not represent standing variation in other areas of the distribution.

Evolutionarily significant units (ESUs^106,107^) are often used to designate lineages of conservation importance. Kaus et al.^46^ proposed two taimen ESUs across northern Mongolia that largely correspond to our Selenge (Arctic) and Amur (Pacific) basin sites. Our results indicating the presence of substantial genetic divergence among these basins lend support for two separate units that might require separate conservation and management strategies. The hierarchical population structure recovered in this study suggests that ecologically relevant genetic variation might be partitioned at smaller spatial scales than previously considered, and we thus caution against the translocation of taimen among geographically and ecologically distinct populations before more thorough genetic sampling can be completed. The differentiated groups we detected in the Tugur basin could reflect ecological and historical variation warranting unique conservation consideration, though further study is clearly needed. Indeed, the additional evidence for population structure within the Pacific drainages suggests that our understanding of taimen genetic structure across different riverscapes is likely limited by both the geographic and genomic extent of sampling. Due to difficulty in sampling via fly fishing, our sampling was opportunistic, geographically sparse, and less than ideal for quantifying how environmental and hydrological variation may influence spatial genetic structure across the distribution. Future studies with more comprehensive sampling within and across basins could be essential for developing a finer scale understanding of the factors influencing evolutionary history of taimen across Siberia and the rest of its range.

## Supplementary Information

Supplementary Information.

## Data Availability

The datasets generated for this study are available at the Dryad Digital Repository (doi: https://doi.org/10.5061/dryad.wstqjq2kd; https://doi.org/10.5061/dryad.wstqjq2kd) and NCBI’s Short Read Archive (accession PRJNA745962; https://dataview.ncbi.nlm.nih.gov/object/ PRJNA745962).
