# Supplementary material for: Hierarchical genetic structure and implications for conservation of the world’s largest salmonid, Hucho taimen
Source: Sci Rep. 2021 Oct 15;11:20508. doi: 10.1038/s41598-021-99530-3 (PMC8520000; doi:10.1038/s41598-021-99530-3)
Supplement: Supplementary file 1 — Supplementary Information. [file 41598_2021_99530_MOESM1_ESM.pdf]

Hierarchical genetic structure and implications for conservation of the world's largest salmonid, *Hucho taimen*

*Lanie M. Galland[1,2], James B. Simmons[1,2], Joshua P. Jahner[2], Agusto R. Luzuriaga-Neira[2], Matthew R. Sloat[3], Sudeep Chandra[1,2,4], Zeb Hogan[2,4], Olaf P. Jensen[5], & Thomas L. Parchman[1,2]

[1]Graduate Program in Ecology, Evolution, and Conservation Biology, University of Nevada, Reno, Reno, NV, USA

[2]Department of Biology, University of Nevada, Reno, Reno, NV, USA

[3]Wild Salmon Center, Portland, OR, USA

[4]Global Water Center, University of Nevada, Reno, Reno, NV, USA

[5]Center for Limnology, University of Wisconsin - Madison, Madison, WI, USA

***Author for correspondence:** Lanie M. Galland, lgalland@unr.edu

Supplementary Information

Supplementary Methods

We conducted a phylogenetic analysis of all sampling localities based on a concatenated alignment of ddRADseq loci using a maximum likelihood approach based. Given patterns of hierarchical genetic structure in previous analyses, and to produce an illustrative tree without an unnecessarily large number of tips, we subsampled 3 individuals from each sampling locality for this analysis. We additionally included three individuals of *Brachymystax lenok* (a closely related salmonid, also sampled in Mongolia) as an outgroup. We generated a multiple alignment of sequenced ddRADseq loci using `ipyrad v. 0.9.15`[1]. Default values were chosen for most parameters, unless otherwise stated below. Nucleotide sites with phred scores less than 33 were considered missing and were replaced with "N," representative of an ambiguous nucleotide base. We began with *de novo* assembly using `vsearch`[2] with a clustering similarity threshold (*clust_threshold*) of 0.85. To account for uneven sequence depth, statistical (*mindepth_statistical*) and majority-rule (*mindepth_majrule*) base calling were set to 5 and 4, respectively. Contigs were reduced to consensus sequences within each individual at each site, and sequences with more than 5% heterozygous bases (*max_Ns_consens*) or more than 8% heterozygous sites (*max_Hs_consens*) were discarded. Next, the clustering step was repeated using identical parameters, but across, rather than within, all individuals. Clusters were then filtered and discarded if they contained more than eight indels (*max_Indels_locus*) or if more than 20% of sites were variable (*max_SNPs_locus*). Finally, we retained all loci found in more than 21 individuals (*min_samples_locus*).

We used the resultant `phylip` output file from `ipyrad` as input for phylogenetic analyses using a maximum likelihood approach. We inferred a maximum likelihood phylogeny used `RAxML v. 8.2.12`[3] using the "-f a" option, which searches for the best-scoring tree and performs a bootstrap analysis. We conducted searches using the GTR + GAMMA evolutionary model of sequence evolution, and the number of bootstrap replicates was assessed using the `autoMRE` option, resulting in 1,000 replicates. Although bootstrap support was low for many nodes at more

recent scales, we rendered the tree in Fig. S2 to show all nodes in order to illustrate relevant patterns of divergence and support across the sampled localities.

Supplementary Tables and Figures

Table S1. DIC values for 5 replicate `entropy` runs of each *k* ancestral model for both the full dataset and the Pacific subset. Mean and standard deviation (SD) are included for each model. Lower DIC scores represent better model fit.

	k	1	2	3	4	5	mean	SD
**Full dataset**	2	2,563,950	2,574,427	2,556,251	2,564,459	2,542,892	2,560,396	11,721
	3	3,397,146	3,389,373	3,417,252	3,453,044	3,465,691	3,424,501	33,712
	4	3,363,991	3,257,494	3,420,572	3,348,324	3,379,798	3,354,036	60,297
	5	3,361,903	3,786,156	3,844,955	3,863,475	3,827,988	3,736,895	211,567
	6	3,751,559	3,697,491	3,835,392	3,427,559	3,998,918	3,742,184	209,568
	7	3,664,111	6,597,391	4,099,092	5,211,434	3,300,373	4,574,480	1,339,402
	8	3,531,952	4,009,169	3,711,057	3,885,740	3,542,185	3,736,020	210,277
	9	4,397,436	3,400,362	3,503,357	12,730,622	3,806,935	5,567,742	4,022,938
**Pacific subset**	2	1,409,122	1,362,080	1,388,500	1,371,819	1,393,363	1,384,977	18,460
	3	1,641,797	2,423,495	1,847,685	1,874,910	2,097,337	1,977,045	297,214
	4	1,623,020	1,567,422	1,487,859	1,661,693	1,557,531	1,579,505	66,476
	5	2,152,078	2,219,632	2,222,038	1,965,042	1,639,002	2,039,558	247,176
	6	2,126,383	2,191,929	1,656,468	2,765,256	1,593,665	2,066,740	474,151

Table S2. Pairwise estimates of mean F_{ST} (upper diagonal) and Nei's *D* (lower diagonal) among all sampling sites. Site abbreviations correspond to those in Table 1.

	DL	EG	UR	UO	LO	KO	MO	AJ	TU
DL	-	0.009	0.006	0.213	0.224	0.255	0.250	0.247	0.258
EG	0.0014	-	0.008	0.209	0.221	0.252	0.247	0.244	0.256
UR	0.0004	0.0008	-	0.212	0.223	0.255	0.250	0.248	0.256
UO	0.1441	0.1405	0.1438	-	0.017	0.039	0.038	0.026	0.036
LO	0.1492	0.1474	0.1485	0.0035	-	0.034	0.034	0.023	0.031
KO	0.1797	0.1767	0.1794	0.0155	0.0109	-	0.029	0.017	0.023
MO	0.1702	0.1698	0.1698	0.0128	0.0085	0.0039	-	0.018	0.022
AJ	0.1736	0.1709	0.1734	0.0093	0.0060	0.0041	0.0027	-	0.012
TU	0.1823	0.1804	0.1797	0.0153	0.0107	0.0042	0.0031	0.0033	-

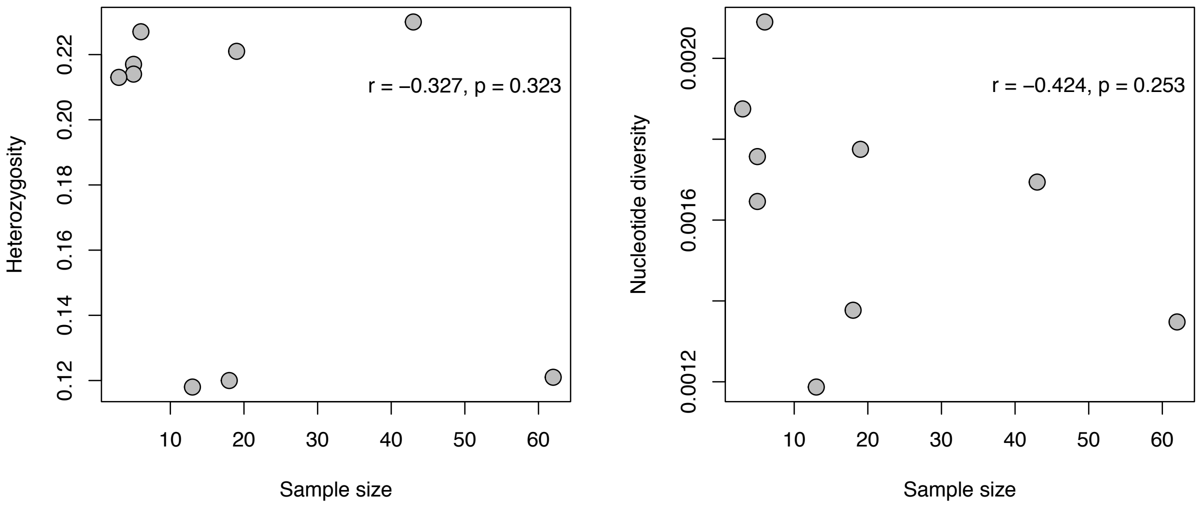

Figure S1. Plots of genetic diversity metrics (heterozygosity and nucleotide diversity) by sample size for each locality. Sample size does not influence estimates of genetic diversity.

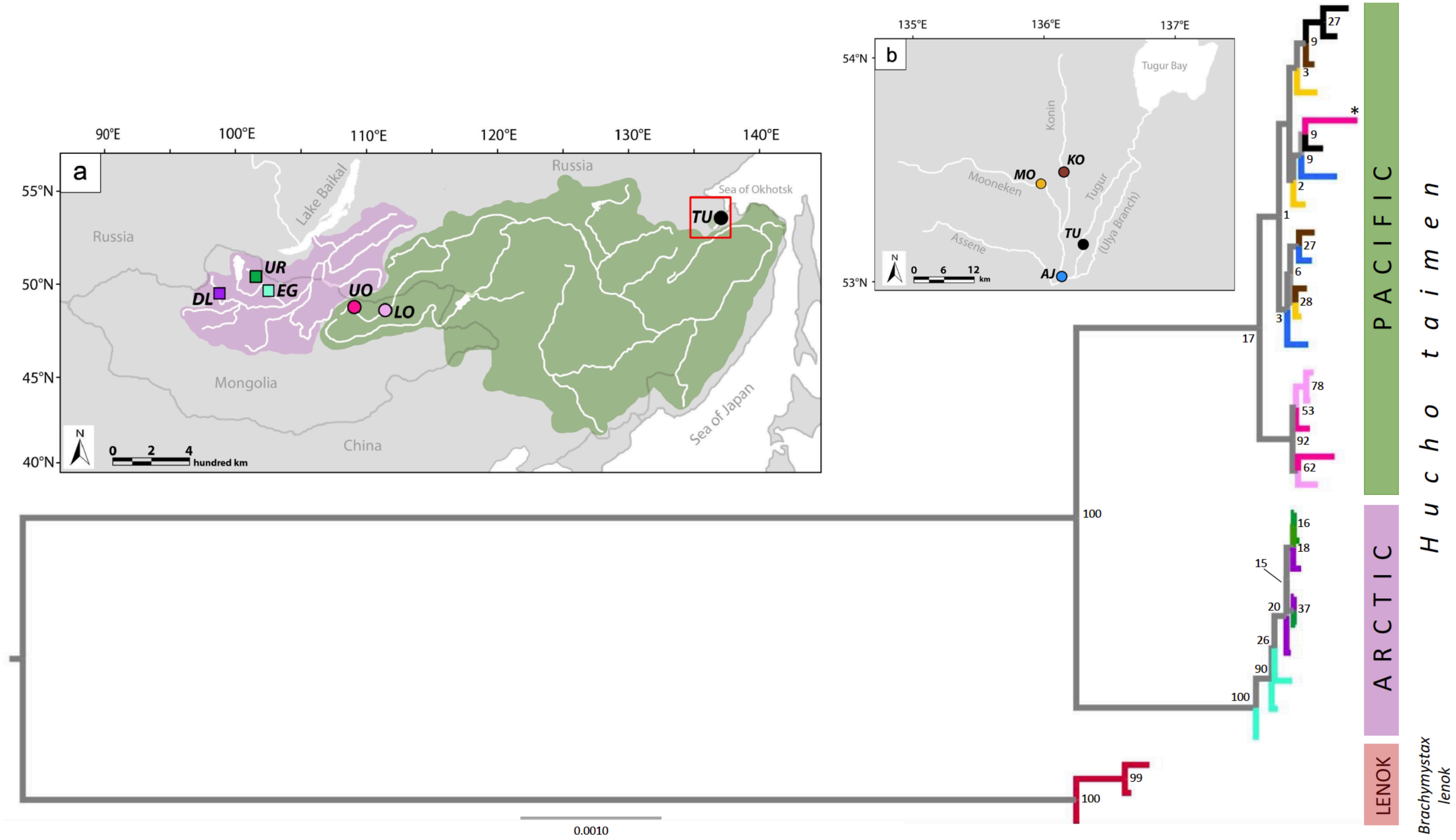

Figure S2. Phylogenetic tree inferred from a concatenated alignment (1,229 RADseq loci, 1,642 phylogenetically informative sites) using RAxML. Bootstrap support (out of 100) is illustrated at nodes. *Brachymystax lenok* was used as an outgroup.

Supplementary References

1. Eaton, D. A. R. PyRAD: assembly of de novo RADseq loci for phylogenetic analyses. *Bioinformatics* btu121 (2014).

2. Rognes, T., Flouri, T., Nichols, B., Quince, C. & Mahé, F. VSEARCH: a versatile open source tool for metagenomics. *PeerJ* **4**, e2584 (2016).

3. Stamatakis, A. The RAxML v8. 2. X manual. Heidleberg Institute for Theoretical Studies. (2016).
